# Supplementary material for: In situ enzymatic control of colloidal phoresis and catalysis through hydrolysis of ATP
Source: Nat Commun. 2024 Apr 29;15:3603. doi: 10.1038/s41467-024-47912-2 (PMC11059368; doi:10.1038/s41467-024-47912-2)
Supplement: Supplementary file 1 — Supplementary Information [file 41467_2024_47912_MOESM1_ESM.pdf]

Supplementary Information

In situ enzymatic control of colloidal phoresis and catalysis through hydrolysis of ATP

Ekta Shandilya, [a] Bhargav Rallabandi,*[b] Subhabrata Maiti*[a]

[a] Department of Chemical Sciences, Indian Institute of Science Education and Research (IISER) Mohali, Knowledge City, Manauli 140306 (India).

[b] Department of Mechanical Engineering, University of California, Riverside, California, 92521, USA.

*Corresponding author

Email: smaiti@iisermohali.ac.in ; bhargav@engr.ucr.edu

Supplementary Table of Contents

1. Supplementary Methods S3
2. Supplementary Tables 1-4 ……………………………………………………………S8
3. Supplementary Figures 1-36 …………………………………………...……. S11
4. Supplementary References.. S32

1. Supplementary Methods

Materials used:

All commercially available reagents were used as received without any further purification. Cetyltrimethylammonium bromide, silver nitrate, sodium borohydride, ascorbic acid, malachite green, hydrochloric acid, calcium nitrate, and ammonium molybdate were procured from Sisco Research Laboratory (SRL), India. Gold (III) chloride trihydrate, adenosine triphosphate sodium salt (ATP), adenosine diphosphate (ADP) sodium salt, adenosine monophosphate (AMP) sodium salt, Potato Apyrase (PA), and carboxylate functionalized polystyrene beads were purchased from Sigma-Aldrich. Throughout the study, we have used milli-Q water.

UV-Vis studies were performed using Varian Cary 60 (Agilent technologies) spectrophotometer. Total reaction volume in the cuvette was fixed at 1 mL and cuvette of path length 1 cm was used for the entire study.

The fluorescence images of microfluidic channel were collected using Zeiss Axis Observer 7 microscope with AxioCam 503 Mono 3 Mega pixel with ZEN 2 software.

The Transmission Electron Microscopy images were taken using JEOL JEM-F200 microscope.

The Dynamic Light Scattering (DLS) and zeta potential data were recorded on Horiba Scientific Nano Particle Observer (SZ-100V2).

The 2-inlet-1-outlet microfluidic chip (1.7 cm length x 600 µm width x 100 µm height) was procured from Vena Delta.

Demountable quartz cuvette having dimension $35 \times 8.5 \times 0.05$ mm^3 (length × width × height) was procured from Lark Scientific.

Synthesis and Characterization of gold nanorods (GNR)

Gold nanorods has been synthesized as reported in literature.[1] A 5 mL solution of 0.1 M cetyltrimethylammonium bromide (CTAB) and 1 mM $HAuCl_4$ was prepared in milli-Q water, which gave a resultant golden colored solution. To this, ascorbic acid was added (which made the solution colorless), followed by the addition of $AgNO_3$. The final concentrations of ascorbic acid and $AgNO_3$ were 2 mM and 0.15 mM, respectively. Lastly, a solution of 1 mM $NaBH_4$

was prepared freshly and 50 µL of it was added, which made the solution violet in color after some time. The violet color indicated the formation of gold-nanorods.

Filtration of GNR

To remove unbound CTAB and other impurities, GNR solution was first filtered before using further. For this purpose, Sephadex G-25 filter was used. Before using, the column was firstly equilibrated with an ample amount of water.

Characterization of GNR

For initial confirmation, thus formed gold nanorods were characterized using UV-Vis spectrophotometer. The obtained spectrum showed a characteristic peak at 750 nm and 523 nm, which confirmed the formation of gold nanorods.

Fabrication of Bead-GNR complex

To form Bead-GNR (CMB) conjugate, carboxylate modified polystyrene beads having 1 µm mean particle diameter were procured from Sigma Aldrich and were used without further modifications. To fabricate Bead-GNR complex, 4 µL (0.1 mg) of carboxylate modified polystyrene beads (d = 1 µm) were dispersed in 896 µl water, later 100 µL gold nanorods (around 150 pM) were added to it and sonicated for 15 minutes.

For removing unbound gold nanorods, this solution was then centrifuged at 2650 x g for 3 minutes so that bead-GNR conjugate got settled down at bottom. After collecting, Bead-GNR conjugate was redispersed in water for using further. To confirm, thus formed conjugate was investigated using TEM. The zeta potential of thus synthesized bead-GNR conjugate was around 80 ± 5 mV in water.

Calculation of ambipolar diffusivity and diffusiophoretic mobility

The ambipolar diffusivity of a binary salt that dissociates into cations of charge z_+ and diffusivity D_+ and anions of charge z_- (note that z_- is negative) and diffusivity D_- is [Eq. (4) of Wilson et al. 2020] [2]

$$D = \frac{(z_+ - z_-)D_+D_-}{z_+D_+ - z_-D_-}. \qquad (S1)$$

The diffusiophoretic mobility of a charged particle is a combination of an electrophoretic component and a chemiphoretic component. To calculate the mobility, we first define the dimensionless zeta potential $\Psi = \frac{\zeta}{k_B T}$ where k_B is Boltzmann's constant, and then use Eq. (8) of Wilson et al. 2020, see also Gupta et al.[2,3] Defining $V_T = k_B T/e$, where e is the electronic charge, the diffusiophoretic mobility is defined by

$$\Gamma_p = \frac{\varepsilon V_T^2}{\mu}\left(\beta\, \Psi_D + \frac{1}{2}\int_0^{\Psi} \frac{\int_0^s g(r)\, dr}{g(s)}\, ds\right), \qquad \text{where} \quad g(s) = [z_+\,(\exp(-s\, z_-) - 1) - z_-(\exp(-s\, z_+) - 1))]^{\frac{1}{2}}, (S2)$$

and $\beta = (D_+ - D_-)/(z_+ D_+ - z_- D_-)$. The term involving β in (S2) is the electrophoretic component and is driven by local electric fields produced by difference in diffusion rates of anions and cations, whereas the second term in (S2) is the chemiphoretic contribution.

We calculate both D and Γ_p values used in the main text under the assumption of NaAMP, Na_2ADP and Na_3ATP salts (with the diffusivities of the species given in Supplementary Table 1), and using the experimentally measured zeta potentials. For typical experimental values, the electrophoretic component is somewhat greater than the chemiphoretic contribution, though both contributions are generally important and the details depend on the salt used. We list ambipolar diffusivities, diffusiophoretic mobilities and the relative contributions of electro- and chemi-phoresis for different nucleotide salts in Supplementary Table 2.

For single nucleotides, the small-potential Debye-Huckel theory (quadratic in the potential) is within 1% of (S2) for the experimentally relevant range of zeta potentials. We exploit this feature to model phoresis in a mixture of ATP and AMP, where we first assume that each nucleotide salt is transported independently (we neglect cross-diffusion terms). We expect this to be a good approximation since the molecular (ambipolar) diffusivities of each nucleotide salts is similar to within 10%. We then compute the diffusiophoretic velocity of the particle

using the small-potential theory given by Eq. (5) of Chiang and Velegol; see also Velegol et al. 2016.[4,5]

Theoretical modelling in non-continuous flow set up

We model one-dimensional transport of CMB in AMP and ATP in a non-continuous flow setup by using Eq. (1) – (2); see Methods, setting $u = 0$. Nucleotides diffuse towards the left arm (Fig. 3a) where there is a lower concentration, and CMB move up the resulting nucleotide gradient due to phoresis. Supplementary Fig. 19 shows concentration profiles at the end of 5 minutes, assuming that the CMB solution introduced into the left arm acts like a "sink" of nucleotides (zero concentration). As a result, the CMB travel about 0.4 mm farther in AMP than in ATP. With a symmetry condition instead (the nucleotide concentration being held at 0.5 mM at the left arm; note that the nucleotide concentration to the far right is 1 mM), the difference in drift is 0.3 mm. These estimates are consistent with the experimentally observed range (Fig. 3).

Measurement of Potato Apyrase activity

To estimate the amount of ATP cleaved during potato apyrase (PA) activity malachite green assay was used. [6] Firstly, phosphate calibration was made using this assay (Figure S27). Then, ATP (0 - 40 µM) was incubated with 100 nM PA along with 0.25 mM Ca^{2+}, and the amount of phosphate formed was measured at different time points. After that, initial activity rate for each set was plotted against ATP concentration and curve fitted into Michaelis–Menten kinetics graph (see Supplementary Fig. S24 + S25). Thus, obtained V_{max}, K_M values were 0.15 s^{-1}, 13.13 µM, respectively.

Kemp Elimination Activity of CMB

NBI (5-nitrobenzisoxazole) and CNP (2-cyano nitrophenol) were synthesized by following previously reported protocols.[7]

The activity of above formed CMB complex was checked for NBI. For sample preparation, above formed CMB complex was treated with NBI (100 µM) in presence of both nucleotides, and mixture of nucleotide (whenever mentioned in manuscript). The amount of CNP ($\varepsilon_{380\ nm}$ = 8000 $M^{-1}cm^{-1}$) formation was quantified by measuring absorbance at 380 nm over time using UV-vis spectrophotometer. The 10 mM stock solution of NBI and CNP were prepared in acetonitrile (ACN), and dimethyl sulfoxide (DMSO), respectively.

2. Supplementary Tables

Supplementary Table 1. Ionic Diffusion coefficient of Nucleotides and sodium ion from literature.[8-11]

S. No.	Component	Diffusion coefficient (m^2/s)
1.	ATP	4×10^{-10}
2.	ADP	4.5×10^{-10}
3.	AMP	5×10^{-10}
4.	Na^+	1.33×10^{-9}

Supplementary Table 2. Calculated ambipolar diffusivities and diffusiophoretic mobilities, showing the relative contributions of electrophoresis and chemiophoresis to the mobility.

	AMP	ADP	ATP
ζ (mV)	47	40	9
D (10^{-10} m^2/s)	7.27	8.06	8.42
Γ_p (10^{-10} m^2/s)	5.67	4.06	0.656
% Electrophoresis	66.7	69.2	89.5
% Chemiphoresis	33.3	30.8	10.5

Supplementary Table 3. Diffusiophoretic drift of CMB inside microfluidic chip while calculated 1.6 cm distant from inlet in presence of different nucleotides. Noted drift of CMB in presence of nucleotide is after subtracting from water. Experimental condition: 0.1 mg/mL bead, [GNR] = 150 pM, [nucleotide] = 1 mM in water at 25 °C.

S. No.	Sample	Diffusiophoretic drift from 4 trials (y/w)	Average ± S.D. (μm)
1.	Water	0.006	
		0.006	
		0.005	
		0.007	
2.	1 mM ATP	0.027	13.01 ± 5.18
		0.034	
		0.034	
		0.015	
3.	1 mM ADP	0.107	57.15 ± 3.54
		0.094	
		0.105	
		0.099	
4.	1 mM AMP	0.102	62.89 ± 4.82
		0.113	
		0.120	
		0.110	

Supplementary Table 4. Diffusiophoretic drift of CMB inside microfluidic chip while calculated 1.6 cm distant from inlet in presence of different ratios of ATP and AMP while keeping total nucleotide concentration constant. Noted drift of CMB in presence of nucleotide is after subtracting from water. Experimental condition: 0.1 mg/mL bead, [GNR] = 150 pM in water at 25 °C.

S. No.	χ_{ATP} ([ATP]/([AMP]+[ATP]))	Diffusiophoretic drift from 4 trials (y/w)	Average ± S.D. (μm)
1.	0	0.102	62.89 ± 4.82
		0.113	
		0.120	
		0.110	
2.	0.001	0.082	49.2 ± 3.43
		0.085	
		0.090	
		0.095	
3.	0.01	0.064	33.72 ± 2.05
		0.058	
		0.061	
		0.065	
4.	0.25	0.037	20.10 ± 1.50
		0.039	
		0.039	
		0.043	
5.	0.5	0.031	14.4 ± 3.46
		0.023	
		0.029	
		0.037	
6.	0.75	0.033	13.95 ± 2.1
		0.031	
		0.025	
		0.028	
7.	1	0.027	13.01 ± 5.18
		0.034	
		0.034	
		0.015	

3. Supplementary Figures

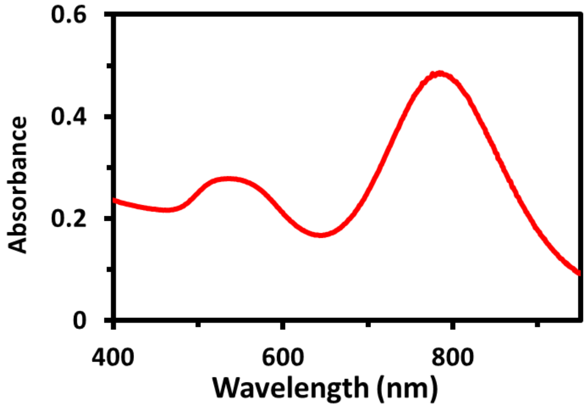

Supplementary Figure 1. UV spectra of GNR. UV spectrum of GNR in mQ water**.** Two spectral maxima at 525 and 780 nm confirms the formation of GNR.

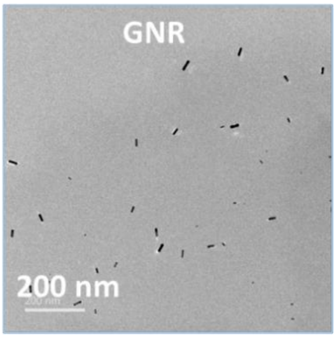

Supplementary Figure 2. TEM image. TEM image of GNR in water. Experimental condition: [GNR] = 150 pM in water at 25 °C.

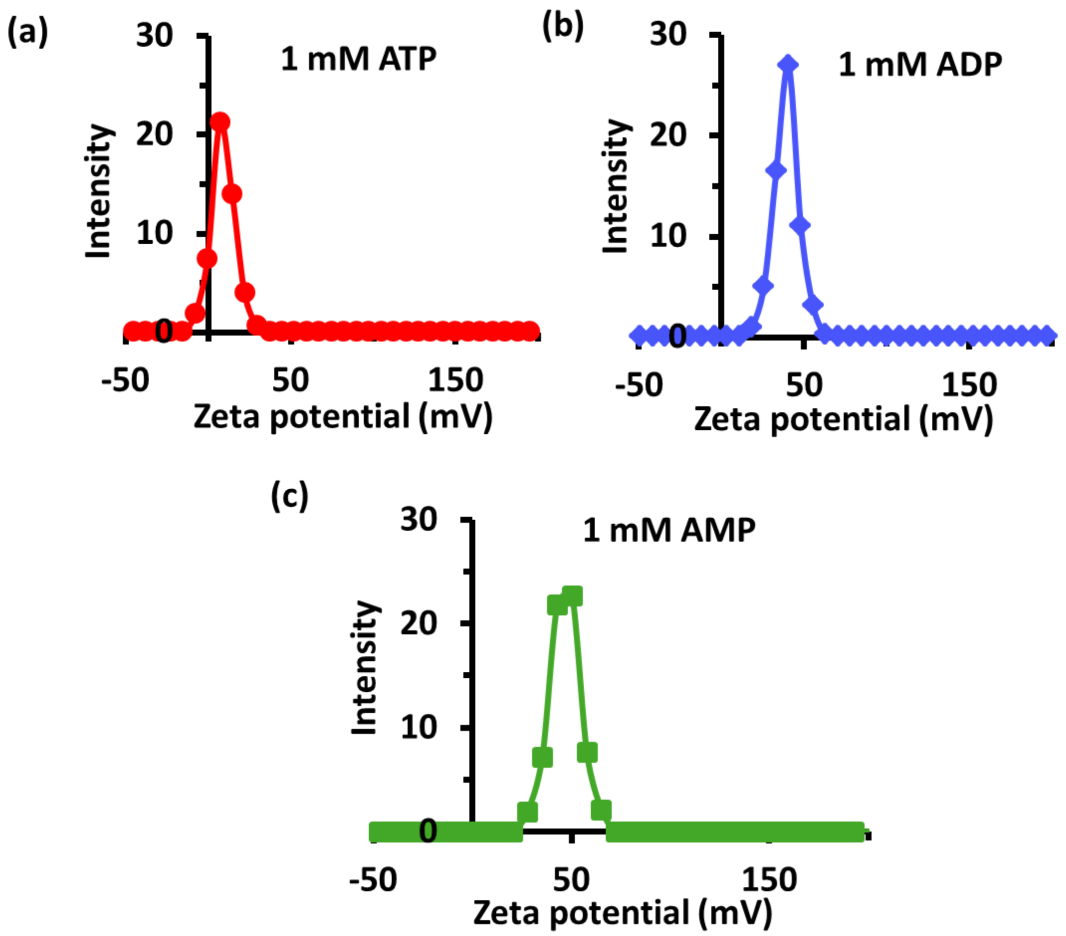

Supplementary Figure 3. Representative zeta potential plot. Zeta potential of CMB in presence of (a) ATP, (b) ADP, (c) AMP. Experimental condition: 0.1 mg/mL bead, [GNR] = 150 pM, [nucleotide] = 1 mM at 25 °C.

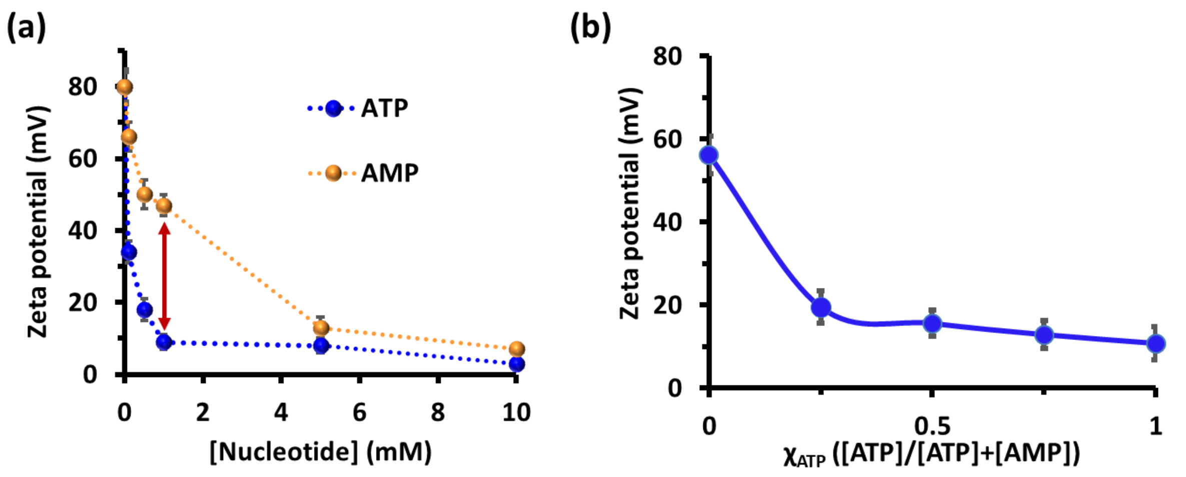

Supplementary Figure 4. Zeta potential measurement. (a) Zeta potential measurement of CMB conjugate in presence of different nucleotide concentrations. Experimental condition: 0.1 mg/mL bead, [GNR] = 150 pM, [nucleotide] = 1 mM at 25 °C. (b) Zeta potential of CMB conjugate in presence different mole fractions of ATP and AMP in presence of NBI. Experimental condition: 0.1 mg/mL bead, [GNR] = 150 pM, [nucleotide] = 1 mM, [NBI] = 0.1 mM at 25 °C.

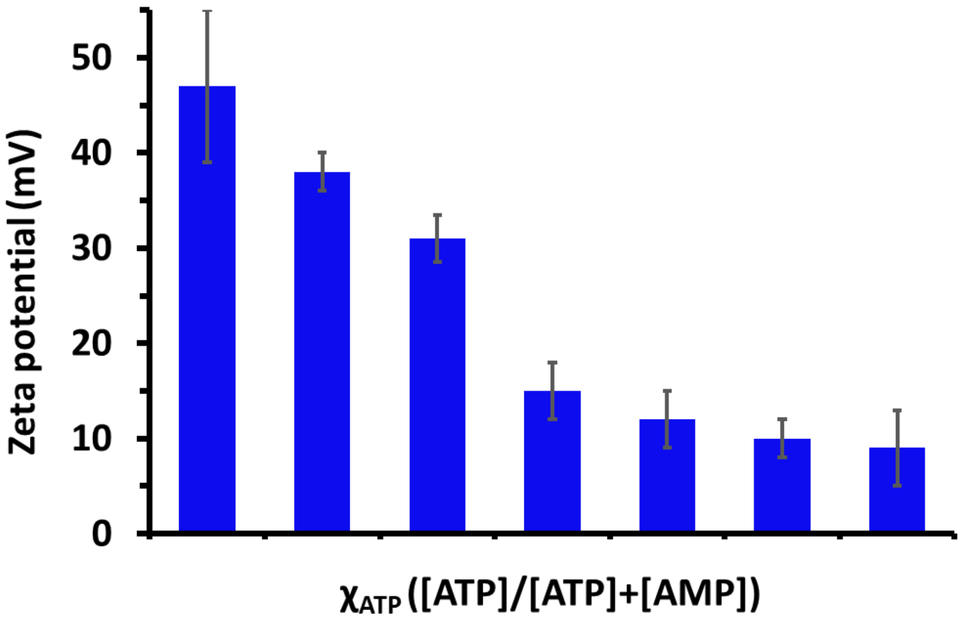

Supplementary Figure 5. Zeta Potential measurement. Zeta potential of CMB in gradient of different composition of AMP and ATP (total nucleotide concentration = 1 mM). Experimental condition: 0.1 mg/mL bead, [GNR] = 150 pM, [nucleotide] = 1 mM at 25 °C.

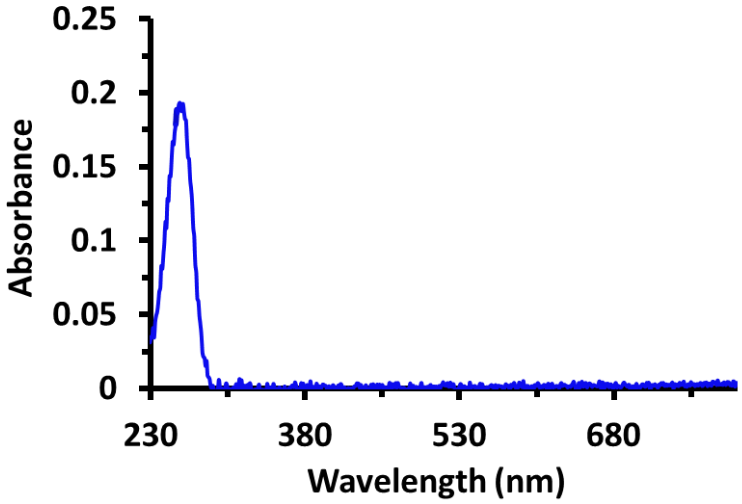

Supplementary Figure 6. Stability of CMB conjugate. UV-vis scan of supernatant of ATP containing CMB solution. Experimental condition: 0.1 mg/mL bead, [GNR] = 150 pM, [ATP] = 1 mM at 25 °C. This experiment ensured the stability of the Bead-GNR conjugate in presence of ATP, 1 mM ATP was added to 1.5 ml vial containing CMB. This sample was centrifuged for 5 minutes at 2650 × g so that all the beads settled at the bottom. UV-vis scan of supernatant showed absence of GNR (Supplementary Fig. S7).

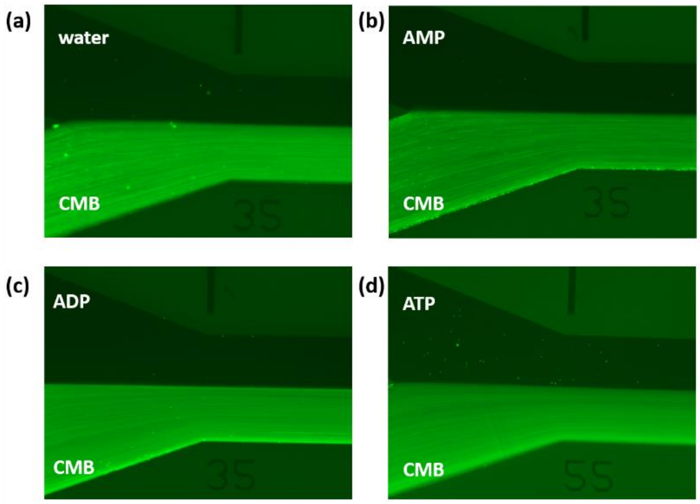

Supplementary Figure 7. Representative fluorescence image of the channel at the inlet. It shows that the drift of the fluorescent CMB showing the interface between two solutions with fluorescent CMB (on the bottom) and only a) water, b) AMP, c) ADP, d) ATP on the top of the channel. Please see Fig. 2a of the main manuscript for the experimental set up. Experimental set up details: Channel width = 0.6 mm, channel height = 0.1 mm, Flow velocity = 300 µl/h. Concentration of nucleotide in each case was 1 mM. The deviation at the inlet with respect to center of the channel and interface of two solutions is less than 3 µm, which is much lower than our observed drift near the outlet (1.6 mm downstream apart) for each case.

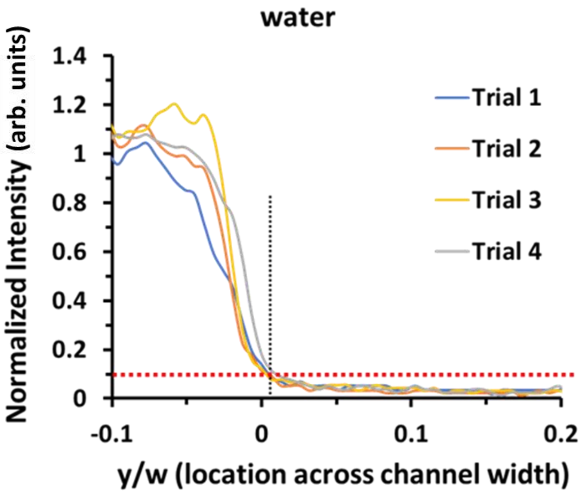

Supplementary Figure 8. Experimental diffusiophoretic drift measurement in microfluidic channel for water as control experiment. Intensity profile of CMB across the microfluidic channel for 4 individual experiments in presence of water. Experimental condition: 0.1 mg/mL beads, [GNR] = 150 pM at 25 °C.

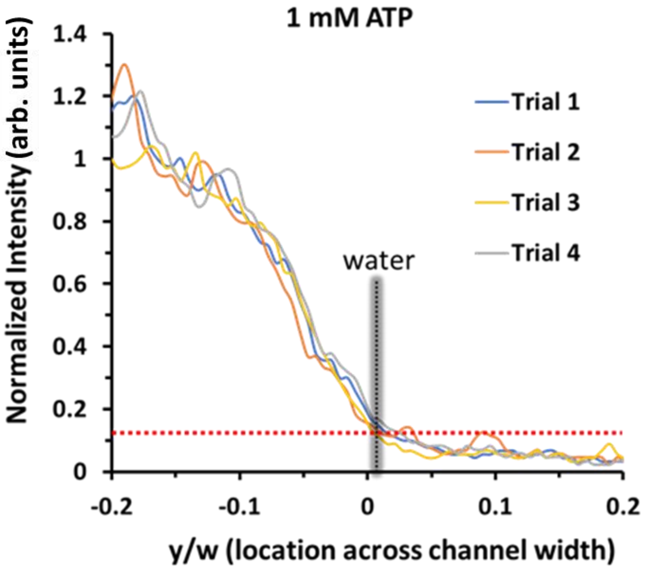

Supplementary Figure 9. Experimental diffusiophoretic drift measurement inside microfluidic channel with ATP. Intensity profile of CMB across the microfluidic channel for 4 individual experiments in presence of ATP. Experimental condition: 0.1 mg/mL beads, [GNR] = 150 pM, [ATP] = 1 mM at 25 °C. Black dotted line serves as guideline to the eye for comparison with control (water case).

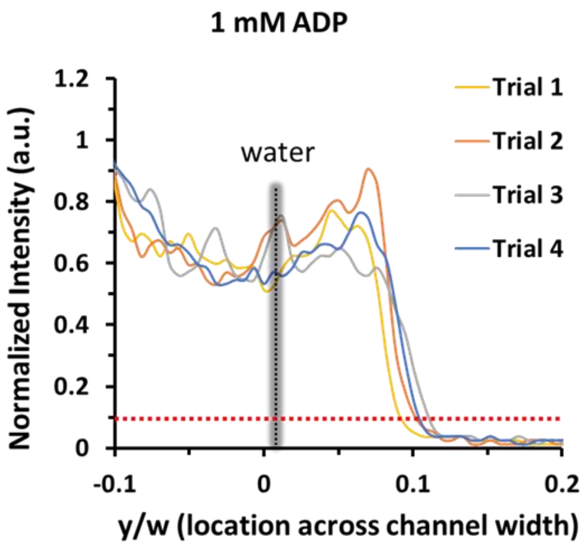

Supplementary Figure 10. Experimental diffusiophoretic drift measurement inside microfluidic channel with ADP. Intensity profile of CMB across the microfluidic channel for 4 individual experiments in presence of ADP. Experimental condition: 0.1 mg/mL beads, [GNR] = 150 pM, [ADP] = 1 mM at 25 °C. Black dotted line serves as guideline to the eye for comparison with control (water case).

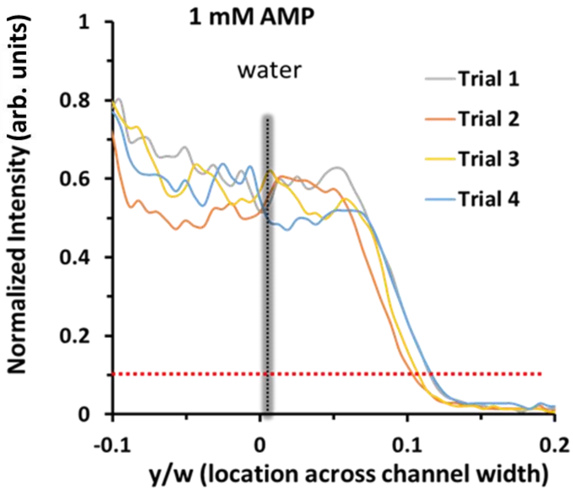

Supplementary Figure 11. Experimental diffusiophoretic drift measurement inside microfluidic channel with AMP. Intensity profile of CMB across the microfluidic channel for 4 individual experiments in presence of AMP. Experimental condition: 0.1 mg/mL beads, [GNR] = 150 pM, [AMP] = 1 mM at 25 °C. Black dotted line serves as guideline to the eye for comparison with control (water case).

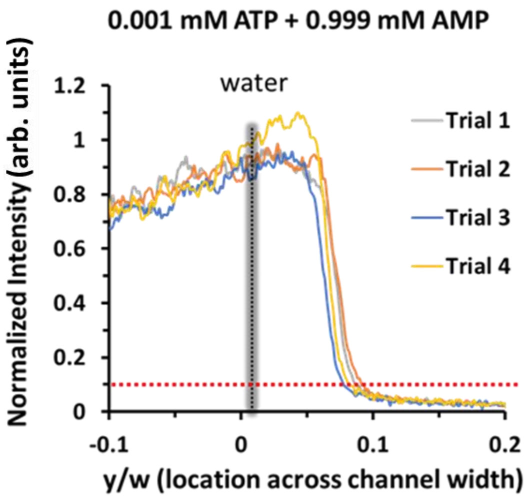

Supplementary Figure 12. Experimental diffusiophoretic drift measurement inside microfluidic channel with a mixture of AMP (0.999 mM) + ATP (0.001 mM). Intensity profile of CMB across the microfluidic channel for 4 individual experiments in presence of ATP and AMP. Experimental condition: 0.1 mg/mL beads, [GNR] = 150 pM, [AMP] = 0.999 mM, [ATP] = 0.001 mM at 25 °C. Black dotted line serves as guideline to the eye for comparison with control (water case).

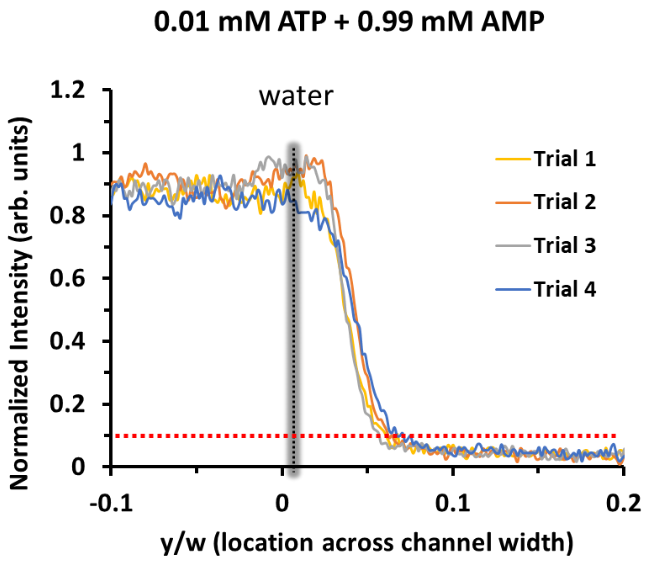

Supplementary Figure 13. Experimental diffusiophoretic drift measurement inside microfluidic channel with a mixture of ATP (0.01 mM) + AMP (0.99 mM). Intensity profile of CMB across the microfluidic channel for 4 individual experiments in presence of ATP and AMP. Experimental condition: 0.1 mg/mL beads, [GNR] = 150 pM, [AMP] = 0.99 mM, [ATP] = 0.01 mM at 25 °C. Black dotted line serves as guideline to the eye for comparison with control (water case).

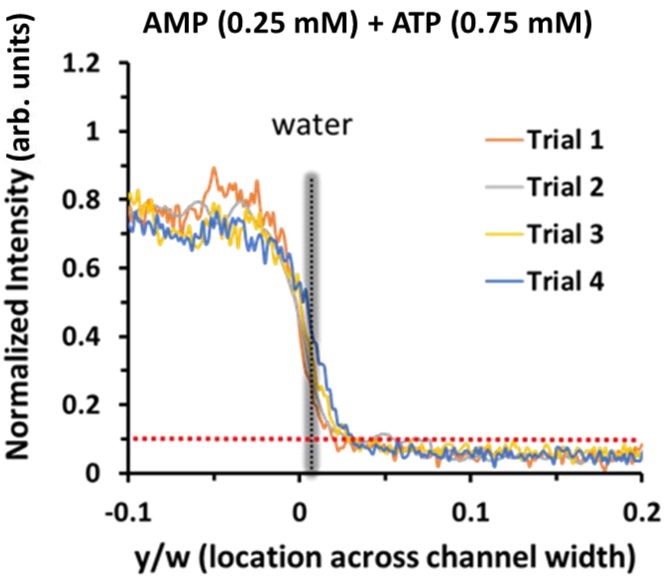

Supplementary Figure 14. Experimental diffusiophoretic drift measurement inside microfluidic channel with a mixture of AMP (0.25 mM) + ATP (0.75 mM). Intensity profile of CMB across the microfluidic channel for 4 individual experiments in presence of ATP and AMP. Experimental condition: 0.1 mg/mL beads, [GNR] = 150 pM, [AMP] = 0.25 mM, [ATP] = 0.75 mM at 25 °C. Black dotted line serves as guideline to the eye for comparison with control (water case).

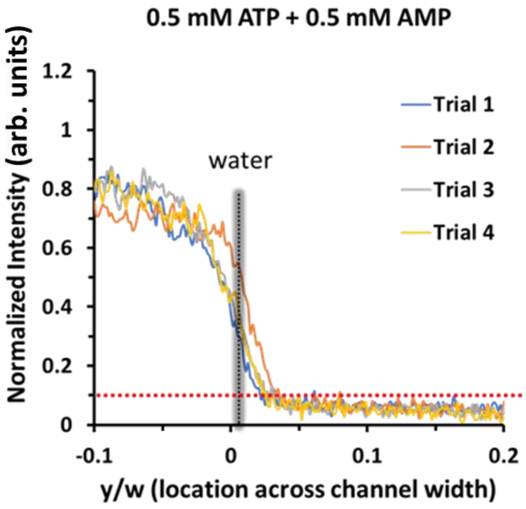

Supplementary Figure 15. Experimental diffusiophoretic drift measurement inside microfluidic channel with a mixture of AMP (0.5 mM) + ATP (0.5 mM). Intensity profile of CMB across the microfluidic channel for 4 individual experiments in presence of ATP and AMP. Experimental condition: 0.1 mg/mL beads, [GNR] = 150 pM, [AMP] = 0.5 mM, [ATP] = 0.5 mM at 25 °C. Black dotted line serves as guideline to the eye for comparison with control (water case).

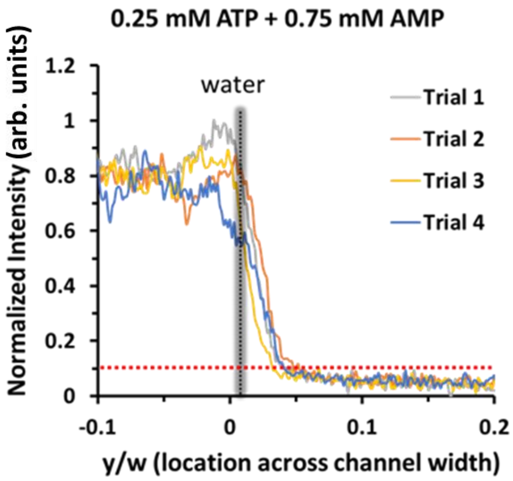

Supplementary Figure 16. Experimental diffusiophoretic drift measurement inside microfluidic channel with a mixture of AMP (0.75 mM) + ATP (0.25 mM). Intensity profile of CMB across the microfluidic channel for 4 individual experiments in presence of ATP and AMP. Experimental condition: 0.1 mg/mL beads, [GNR] = 150 pM, [AMP] = 0.75 mM, [ATP] = 0.25 mM at 25 °C. Black dotted line serves as guideline to the eye for comparison with control (water case).

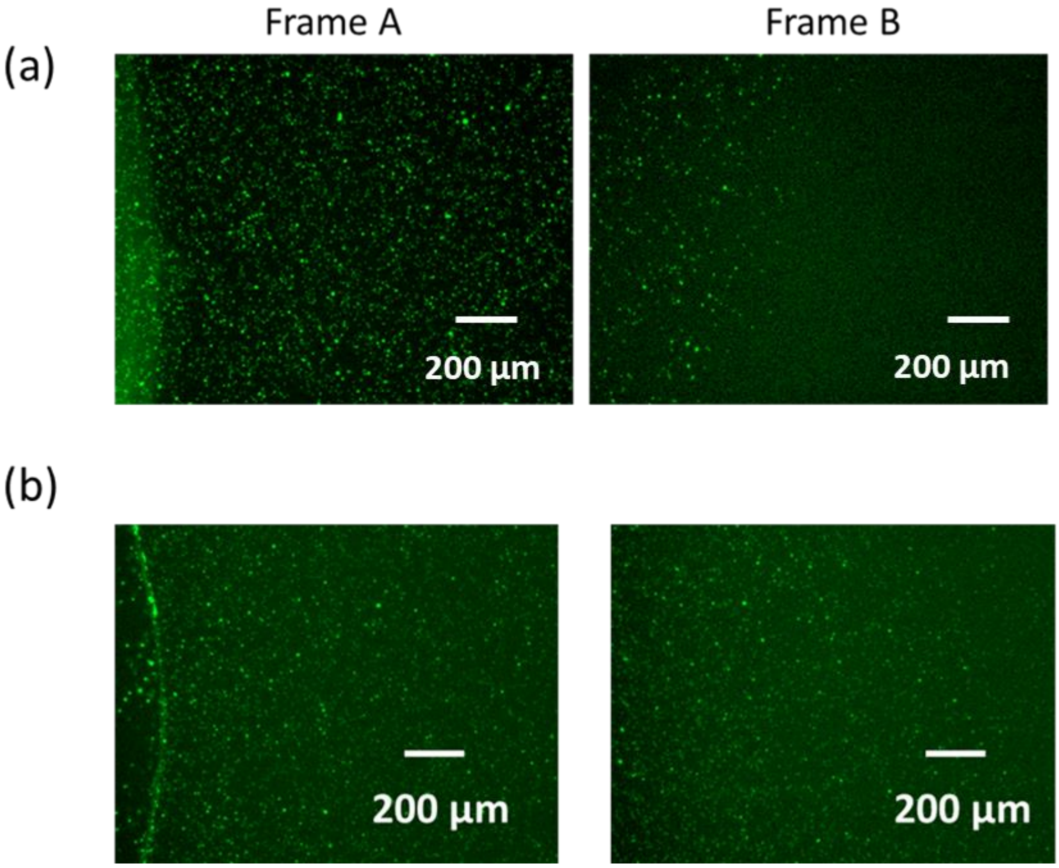

Supplementary Figure 17. Images after 5 min at zone A and B to show the CMB drift in non-continuous flow setup in water only. (a) Migration of CMB inside non-continuous flow chamber in milli-Q only. Experimental condition: 0.025 mg/mL bead, [GNR] = 37.5 pM at 25 °C. X-axis of each image is 1.5 mm. (b) Fluorescence microscopic images of frame A and frame B after 5 minutes of CMB addition showing settling of CMB over surface. Experimental condition: 0.025 mg/mL bead, [GNR] = 37.5 pM, [AMP] = 0.1 mM, [Ca^{2+}] = 0.25 mM at 25 °C.

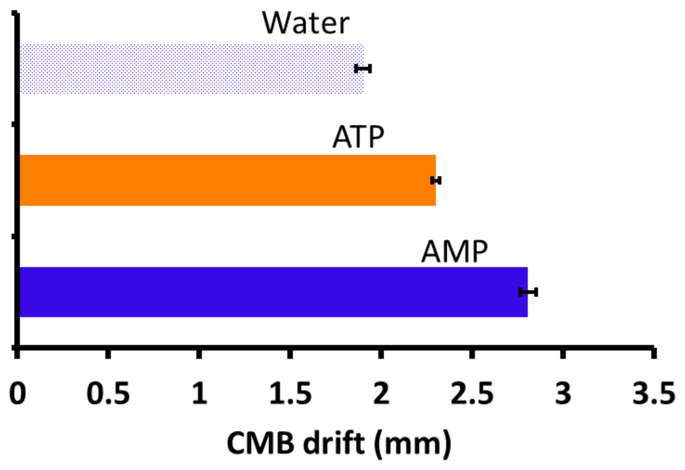

Supplementary Figure 18. CMB drift after 5 min in non-continuous flow setup in presence of nucleotide. Phoretic drift of CMB in non-continuous flow chamber in presence of different nucleotides and water. Experimental condition: 0.025 mg/mL bead, [GNR] = 37.5 pM, [Nucleotide] = 1 mM at 25 °C. The error bar is the standard deviation of 5 experiments.

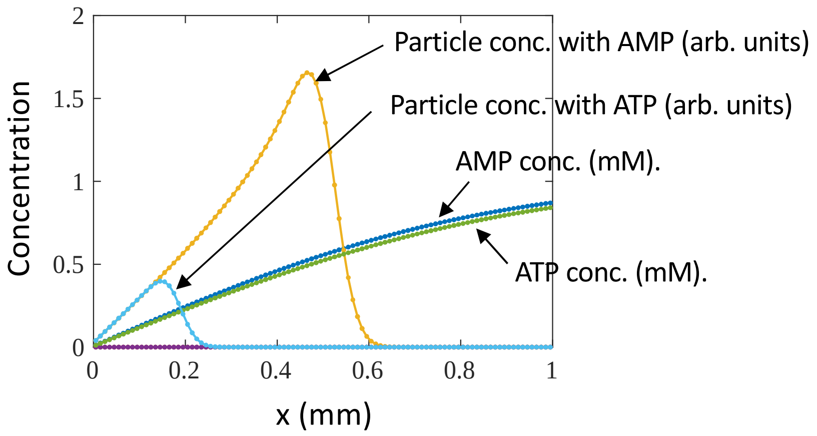

Supplementary Figure 19. Theoretically calculated concentration profile of CMB as a function of distance in gradient of nucleotides. Concentration profiles of nucleotides and particles (CMB) in a non-continuous flow setup after 5 minutes, obtained from solutions to a one-dimensional diffusiophoretic model. Particles drift is about 0.4 mm more in AMP than in ATP.

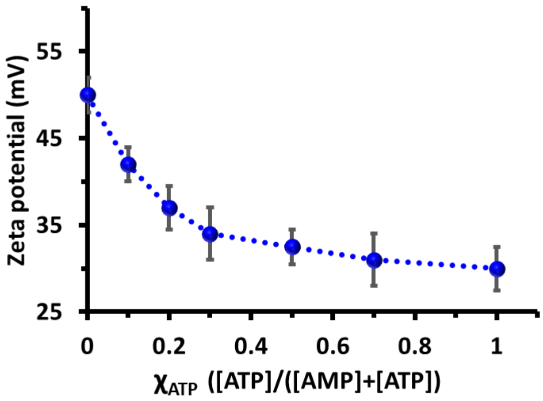

Supplementary Figure 20. CMB zeta potential at different ATP/AMP ratio. Zeta potential of CMB in gradient of different composition of AMP + 2 Pi and ATP (total nucleotide concentration = 10 µM). Experimental condition: 0.025 mg/mL bead, [GNR] = 37.5 pM, $[Ca^{2+}]$ = 0.25 mM at 25 °C.

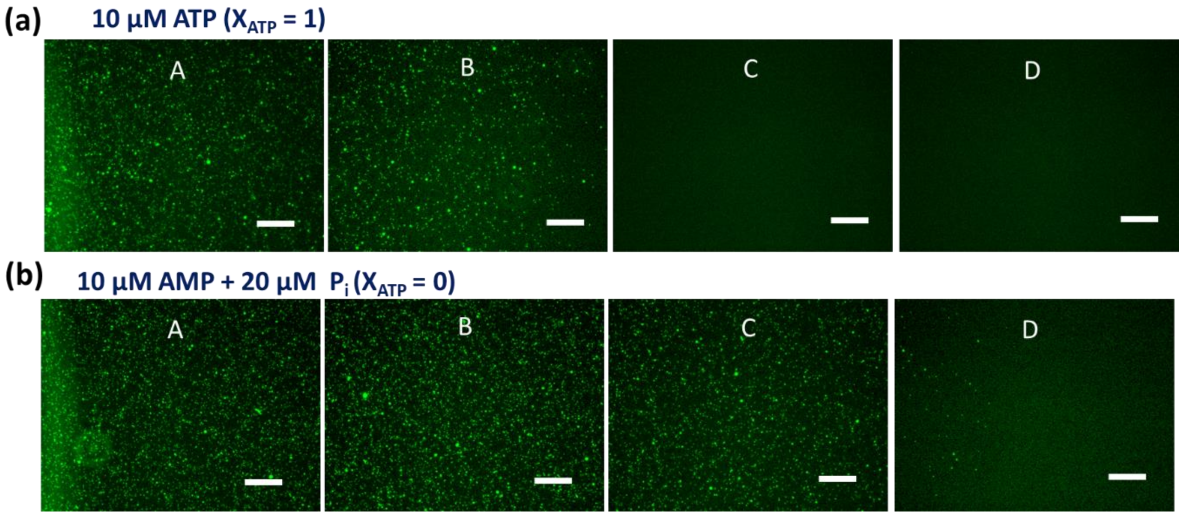

Supplementary Figure 21. Microscopic images of CMB at different zone in ATP and AMP+2Pi gradient. Migration of CMB with (a) ATP, (b) AMP + 2Pi without PA. (c) Additional CMB drifts in reference to water in presence of different ratios of ATP and AMP so that total nucleotide concentration was 10 µM, and amount of Pi was double the concentration of AMP in each case. Scale bar = 200 µm. Experimental condition: 0.025 mg/mL bead, [GNR] = 37.5 pM, [ATP] = 10 µM, [Pi] = 20 µM, [AMP] = 10 µM, $[Ca^{2+}]$ = 0.25 mM at 25 °C.

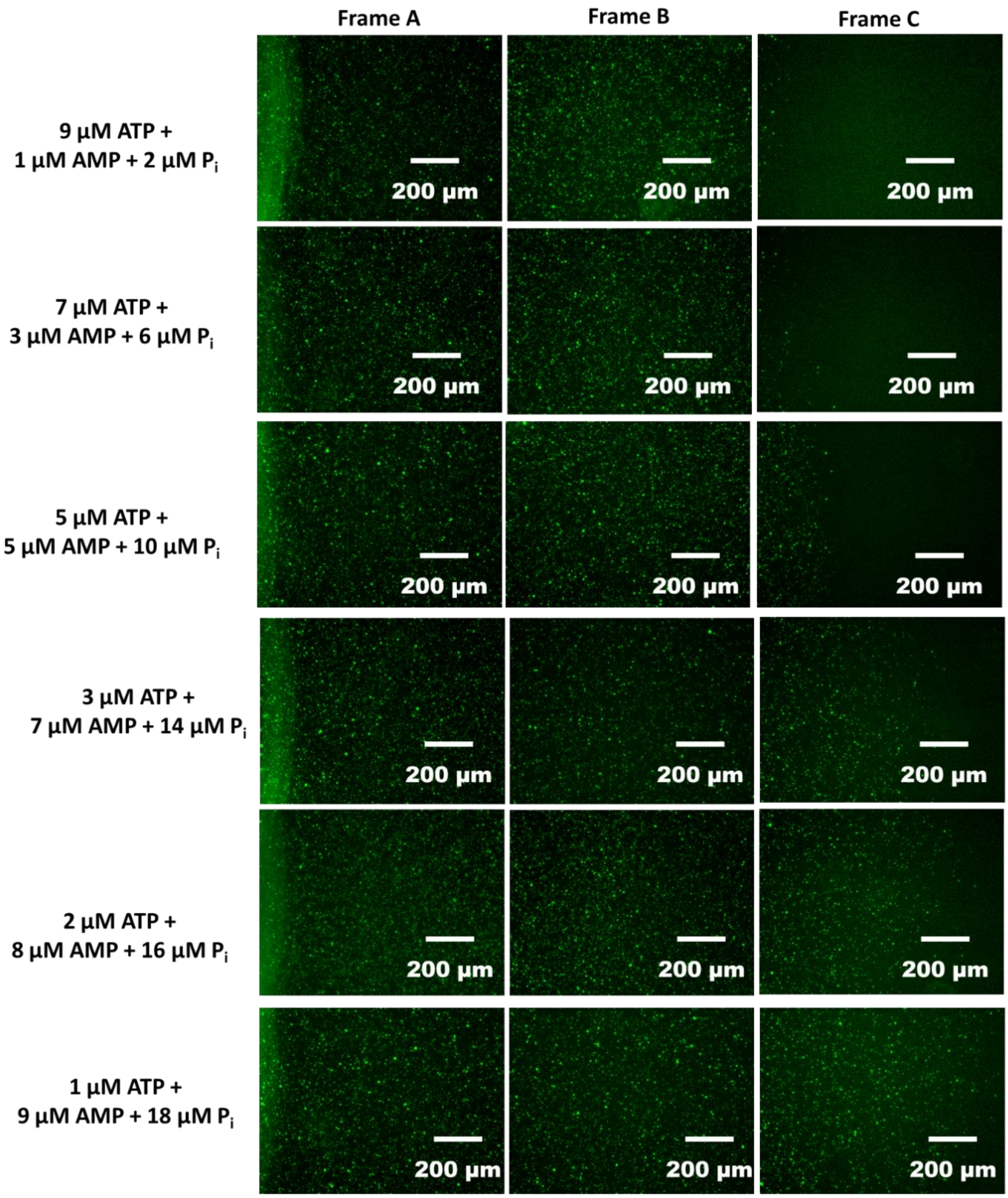

Supplementary Figure 22. Microscopic images of CMB at different zone in different concentration of ATP and AMP+2Pi gradient. Migration of CMB inside non-continuous flow channel while varying concentrations of ATP, AMP and Pi while keeping total concentration constant, that is, 10 µM. Experimental condition: 0.025 mg/mL bead, [GNR] = 37.5 pM , [Nucleotide] = 10 µM, [Pi] = 2 x [AMP], [Ca^{2+}] = 0.25 mM at 25 °C. All the images were taken after 5 min.

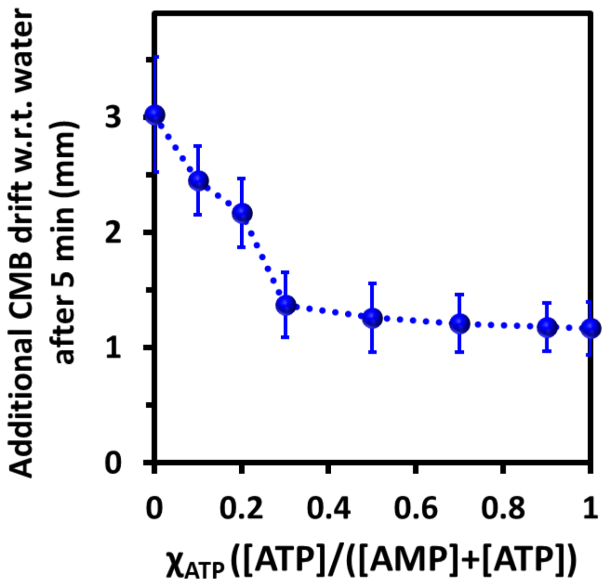

Supplementary Figure 23. Additional CMB drift in reference to water in presence of different ratios of ATP and AMP + 2Pi. In this case total nucleotide concentration (ATP + AMP) was kept constant at 10 µM. Experimental condition: 0.025 mg/mL bead, [GNR] = 37.5 pM, [Nucleotide] = 10 µM, [Pi] = 2 x [AMP], [Ca^{2+}] = 0.25 mM at 25 °C.

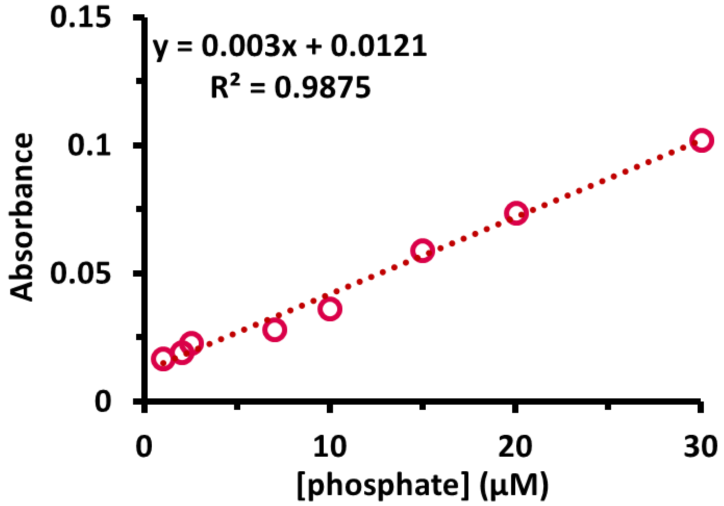

Supplementary Figure 24. Calibration of phosphate using via UV absorbance. Phosphate calibration curve using Malachite green assay.

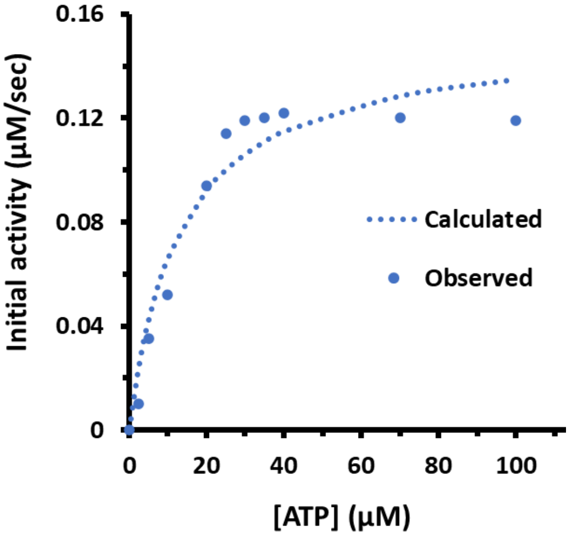

Supplementary Figure S25. Michaelis-Menten Plot. Initial activity measured for phosphate formation plotted against amount of ATP. Blue dotted line denotes Michaelis–Menten plot fitting. The obtained V_{max}, K_M values were 0.15 s^{-1}, 13.13 µM, respectively.

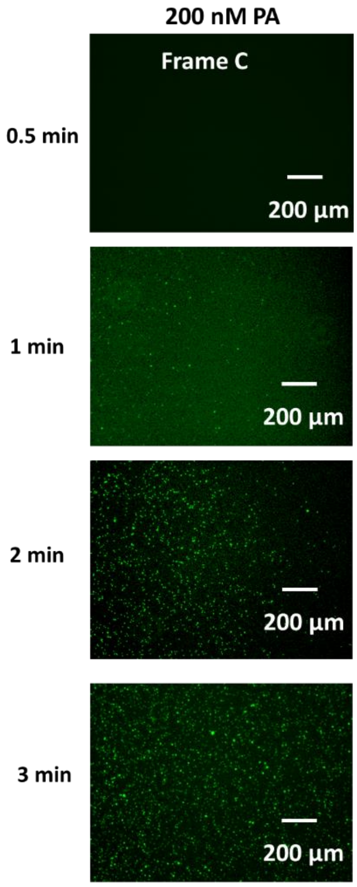

Supplementary Figure 26. Image of CMB at frame C at different time interval. Migration of CMB in Frame C with time in presence of ATP and potato apyrase. Experimental condition: 0.025 mg/mL bead, [GNR] = 37.5 pM, [ATP] = 10 µM, [PA] = 200 nM, [Ca^{2+}] = 0.25 mM in water at 25 °C.

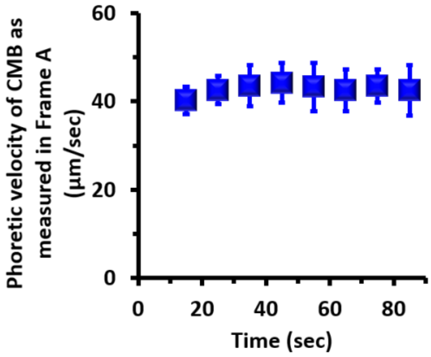

Supplementary Figure 27. Phoretic velocity of CMB in Frame A as a function of tim in presence of ATP and 200 nM PA. Phoretic velocity of CMB measured in frame A during ATP hydrolysis at 250 nM PA concentration. Please see Figure 4 of the main manuscript for experimental set up. Experimental condition: 0.025 mg/mL bead, [GNR] = 37.5 pM, [ATP] =10 µM, [PA] = 250 nM at 25 °C . Channel length = 35 mm, height = 0.05 mm and width = 8.5 mm. the error bar is standard deviation of 2 independent experiments.

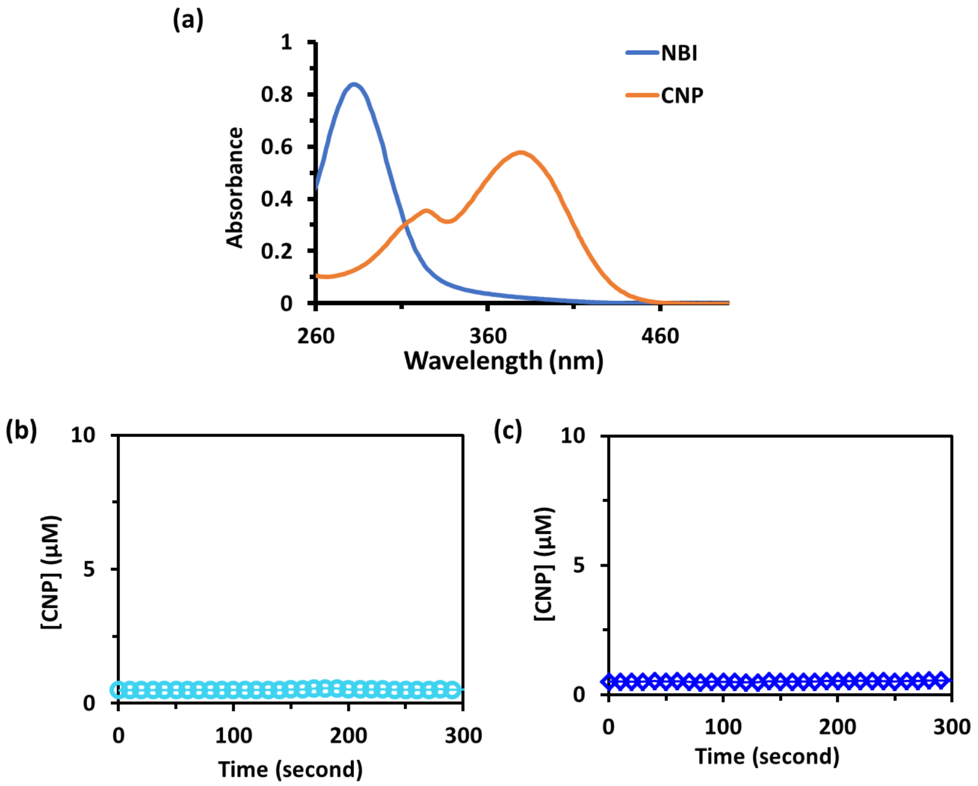

Supplementary Figure 28. UV spectra of KE reactant and product with control experiment to show no product formation in water in absence of GNR and AMP. (a) Scan spectra of NBI and CNP in water. (b) Amount of CNP formation with carboxylate beads (without GNR) in water. Experimental condition: 0.1 mg/mL carboxylate beads, [NBI] = 100 μM in water at 25 °C. (c) Amount of CNP formation with CMB (bead-GNR conjugate) in water. Experimental condition: 0.1 mg/mL beads, [GNR] = 150 pM, [NBI] = 100 μM in water at 25 °C. It demonstrates in absence of nucleotide NBI to CNP conversion does not take place.

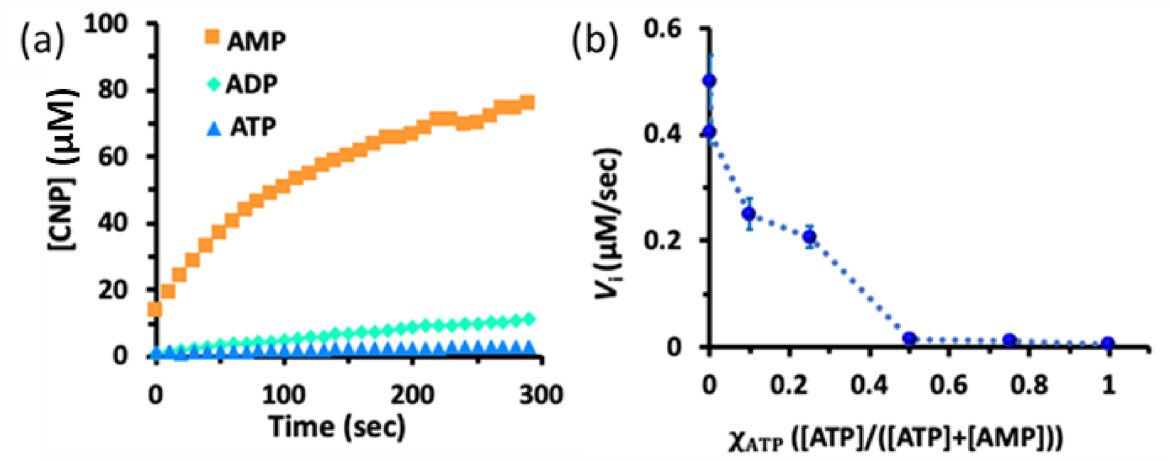

Supplementary Figure 29. KE reaction profile with time and diffent nucleotide content. (a) Amount of Kemp elimination product (2-cyano nitrophenol (CNP)) formed after addition of different nucleotides (1 mM) as a function of time. (b) Initial rate of CNP formation in presence of different fraction of ATP in a mixture of ATP and AMP. Experimental condition: 0.1 mg/mL beads, [GNR] = 150 pM, [NBI] = 100 μM in water at 25 °C. [nucleotide] = 1 mM, for panel (b) total concentration of ATP and AMP is always maintained at 1 mM.

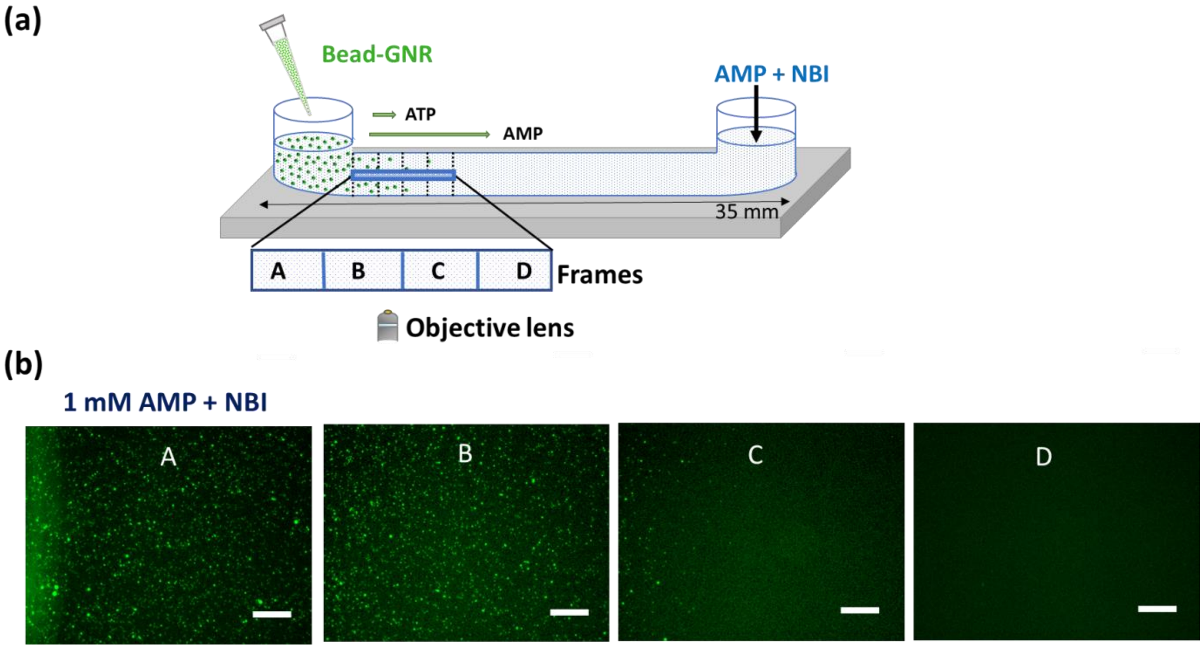

Supplementary Figure 30. Phoretic drift of CMB during KE catalysis in non-continuous flow set up. (a) Schematic representation of non-continuous flow channel while filling change from AMP and NBI solution from one arm, and adding Bead-GNR from the other arm. (b) Migration of Bead-GNR conjugate in different frames. Scale bar = 200 µm. Experimental condition: 0.025 mg/mL bead, [GNR] = 37.5 pM, [AMP] =1 mM, [NBI] = 0.1 mM at 25 °C.

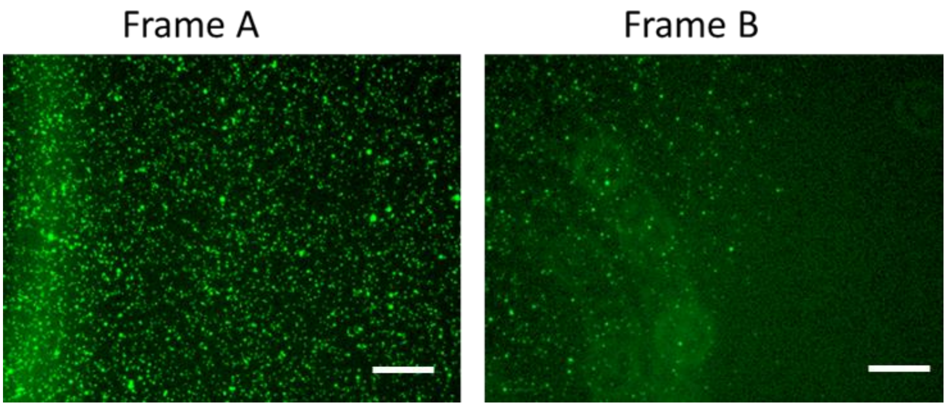

Supplementary Figure 31. Images of CMB during KE catalysis at zone A and B in non-continuous flow set up. Migration of CMB inside non-continuous flow channel in presence of NBI. Scale bar = 200 µm. Experimental condition: 0.025 mg/mL bead, [GNR] = 37.5 pM, [NBI] = 0.1 mM at 25 °C.

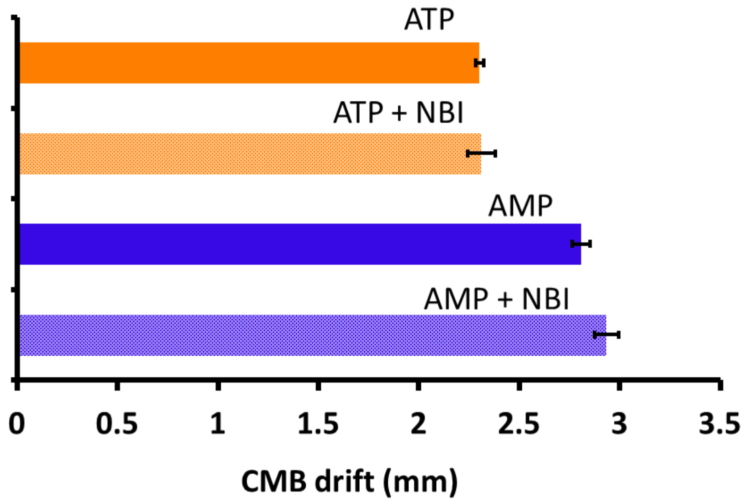

Supplementary Figure 32. Phoretic drift of CMB during KE catalysis in non-continuous flow set up. Phoretic drift of CMB in non-continuous flow chamber in presence of different nucleotides and water. Experimental condition: 0.025 mg/mL bead, [GNR] = 37.5 pM, [Nucleotide] = 1 mM, [NBI] = 1 mM at 25 °C. Due to presence of NBI, the drift does not change significantly, suggesting AMP-mediated diffusiophoresis play the major role in transport.

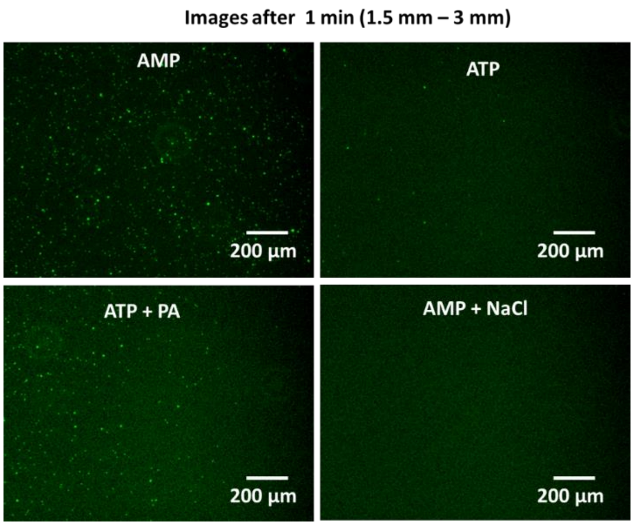

Supplementary Figure 33. Images of CMB during KE catalysis after 1 min in non-continuous flow set up. Migration of Bead-GNR inside non-continuous flow channel 1.5 mm distant from armhole with only nucleotide (ATP and AMP), with AMP + NaCl, and during enzymatic action (ATP to AMP conversion by PA) after 3 minute of Bead-GNR . Experimental condition: 0.025 mg/mL bead, [GNR] = 37.5 pM, [ATP] = 100 µM, [AMP] = 100 µM, [PA] = 500 nM, [NaCl] = 100 µM, [Ca^{2+}] = 0.25 mM, [NBI] =1 mM at 25 °C.

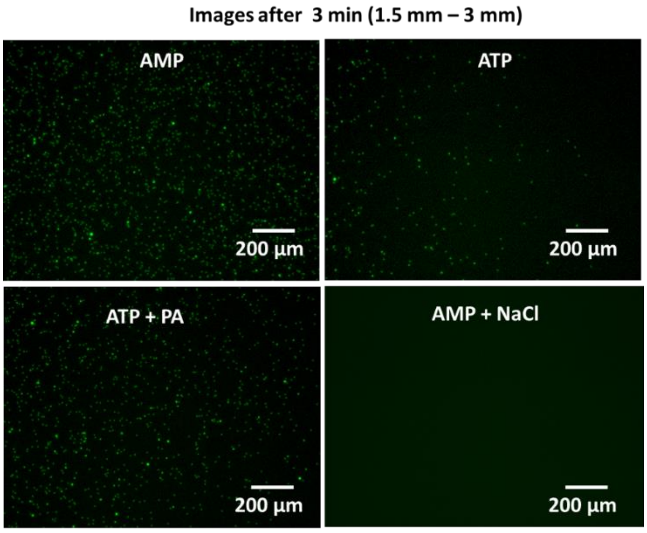

Supplementary Figure 34. Images of CMB during KE catalysis after 3 min in non-continuous flow set up. Migration of Bead-GNR inside non-continuous flow channel 1.5 mm distant from armhole with only nucleotide (ATP and AMP), with AMP + NaCl, and during enzymatic action (ATP to AMP conversion by PA) after 3 minutes of Bead-GNR. Experimental condition: 0.025 mg/mL bead, [GNR] = 37.5 pM, [ATP] = 100 µM, [AMP] = 100 µM, [PA] = 500 nM, [NaCl] = 100 µM, [Ca^{2+}] = 0.25 mM, [NBI] =1 mM at 25 °C.

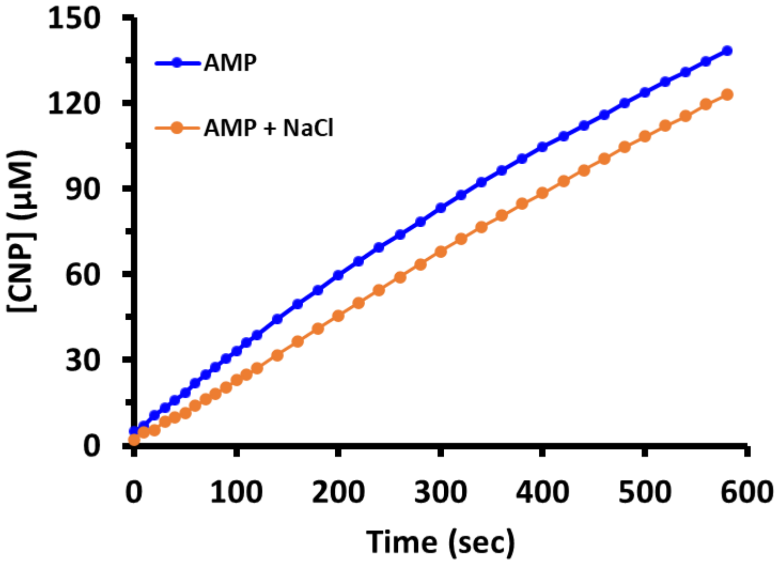

Supplementary Figure 35. KE reaction monitoring with time. Amount of CNP formation with AMP, and AMP + NaCl. Experimental condition: 0.025 mg/mL bead, [GNR] = 37.5 pM, [AMP] = 100 µM, [NaCl] = 100 µM, [NBI] =1 mM at 25 °C.

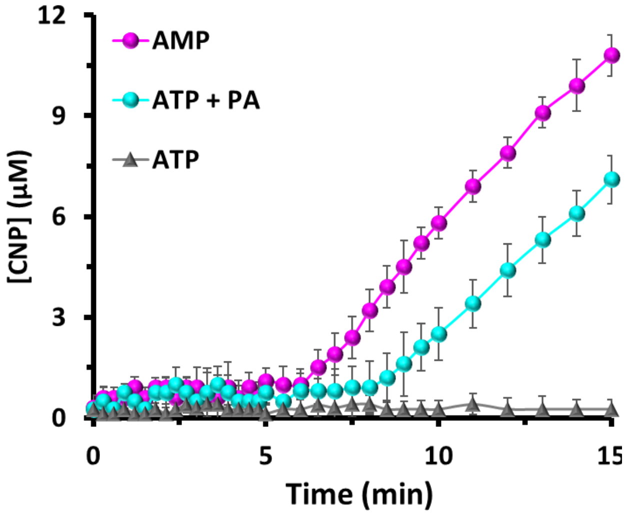

Supplementary Figure 36. KE reaction monitoring with time in non-continuous flow set up at zone 2. Amount of CNP traveled to Zone 2 of non-continuous flow channel solely due to diffusion with only nucleotide (ATP and AMP), and during enzymatic action (ATP to AMP conversion by PA). Experimental condition: 0.025 mg/mL bead, [GNR] = 37.5 pM, [ATP] = 100 µM, [AMP] = 100 µM, [PA] = 500 nM, [Ca^{2+}] = 0.25 mM, [NBI] =1 mM at 25 °C.

4. Supplementary References

1. Maiti, S., Ghosh, M. & Das, P. K. Gold Nanorod in Reverse Micelles: A Fitting Fusion to Cat-apult Lipase Activity. *Chem. Commun* **47**, 9864–9866 (2011).

2. Wilson, J. L., Shim, S., Yu, Y. E., Gupta, A. & Stone, H. A. Diffusiophoresis in multi-valent electrolytes. *Langmuir* **36**, 7014–7020 (2020).

3. Gupta, A., Rallabandi, B. & Stone, H. A. Diffusiophoretic and diffusioosmotic veloci-ties for mixtures of valence-asymmetric electrolytes. *Phys. Rev. Fluids* **4**, (2019).

4. Chiang, T.-Y. & Velegol, D. Multi-ion diffusiophoresis. *J. Colloid Interface Sci.* **424**, 120–123 (2014).

5. Velegol, D., Garg, A., Guha, R., Kar, A. & Kumar, M. Origins of concentration gradi-ents for diffusiophoresis. *Soft Matter* **12**, 4686–4703 (2016).

6. Illingworth, M., Ramsey, A., Zheng, Z. & Chen, L. Stimulating the substrate folding ac-tivity of a single ring GroEL variant by modulating the cochaperonin GroES. *J. Biol. Chem.* **286**, 30401–30408 (2011).

7. Shandilya, E., Dasgupta, B. & Maiti, S. Interconnectivity between surface reactivity and self-assembly of Kemp elimination catalyzing nanorods. *Chem. Eur. J.* **27**, 7831–7836 (2021).

8. Song, Z. *et al.* Characterizing the binding of nucleotide ATP on serum albumin by 31P NMR diffusion. *Can. J. Chem.* **90**, 411–418 (2012).

9. Section, A. *Groundwater Book & Modeling Techniques. Description of input and examples for PHREEQC version 3-A computer program for speciation, batch-reaction, one-dimensional transport, and inverse geochemical calculations*.

10. Dworkin, M. & Keller, K. H. Solubility and diffusion coefficient of adenosine 3':5'-monophosphate. *J. Biol. Chem.* **252**, 864–865 (1977).

11. Lu, H., Ali, M. Y., Bookwalter, C. S., Warshaw, D. M. & Trybus, K. M. Diffusive movement of processive kinesin-1 on microtubules. *Traffic* **10**, 1429–1438 (2009).
