# Supplementary material for: In situ enzymatic control of colloidal phoresis and catalysis through hydrolysis of ATP
Source: Nat Commun. 2024 Apr 29;15:3603. doi: 10.1038/s41467-024-47912-2 (PMC11059368; doi:10.1038/s41467-024-47912-2)
Supplement: Supplementary file 3 — Description of Additional Supplementary Files [file 41467_2024_47912_MOESM3_ESM.pdf]

<u>Description of Additional Supplementary Files</u>

Supplementary Movie 1. To record the movement of CMB inside non-continuous flow setup, videos were recorded in the first frame (0 – 1500 µm) after 10 seconds of CMB addition. Migration of CMB in Frame A with time in presence of ATP only.

Supplementary Movie 2. To record the movement of CMB inside non-continuous flow setup, videos were recorded in the first frame (0 – 1500 µm) after 10 seconds of CMB addition. Migration of CMB in Frame A with time in presence of ATP, and 200 nM PA.
